# Transcriptomic approaches for identifying potential transmission blocking vaccine candidates in *Plasmodium falciparum*: a review of current knowledge and future directions

**DOI:** 10.1007/s13205-023-03752-3

**Published:** 2023-09-12

**Authors:** Gutthedhar Varijakshi, Mallya Divya, Akshay Pramod Ware, Bobby Paul, Abdul Vahab Saadi

**Affiliations:** 1 Department of Biotechnology, Manipal School of Life Sciences, Manipal Academy of Higher Education, Manipal, Karnataka 576104 India; 2 Department of Bioinformatics, Manipal School of Life Sciences, Manipal Academy of Higher Education, Manipal, Karnataka 576104 India

**Keywords:** Gametocyte, Vaccine, Differential expression analysis (DEA), Transition, Adaptation

## Abstract

Utilizing transcriptomics, promising methods for identifying unique genes associated with *Plasmodium* gametocyte development offer a potential avenue for novel candidate targets in transmission blocking vaccine development. In this review, we identified 40 publicly available transcriptomic datasets related to parasite factors linked with sexual stage transmission, from which we analyzed two RNA-Seq datasets to identify potential genes crucial for the transmission of *P. falciparum* from humans to mosquito vectors. Differential expression analysis revealed 3500 (2489 upregulated and 1011 downregulated) common genes differentially expressed throughout sexual stage development of *P. falciparum* occurring in both humans (gametocyte stage II, V) and mosquitoes (ookinete). Among which 1283 (914 upregulated and 369 downregulated) and 826 (719 upregulated and 107 downregulated) genes were specific to female and male gametocytes, respectively. Also, 830 potential transition associated genes were identified that may be involved in the adaptation and survival of the parasite in between human and mosquito stages. Additionally, we reviewed the functional aspects of important genes highly expressed throughout the sexual stage pathway and evaluated their suitability as vaccine candidates. The review provides researchers with insight into the importance of publicly available transcriptomic datasets for identifying critical and novel gametocyte markers that may aid in the development of rational transmission blocking strategies.

**Supplementary Information:**

The online version contains supplementary material available at 10.1007/s13205-023-03752-3.

## Introduction

An estimated 247 million cases of malaria were reported across 84 malaria endemic countries in 2021, indicating a rise of two million cases from the previous year. Although over 50 countries have successfully eradicated malaria, isolated cases still occur due to imported malaria cases from migrants and travellers from endemic regions (World Malaria Report 2022). These cases can harbour the sexual stage parasite, known as gametocytes, which persist in the bloodstream even after treatment and can continue the parasite’s life cycle if taken up by the female *Anopheles* mosquito vector. Therefore, targeting the gametocyte stage could significantly improve the ability to control and eliminate malaria in various transmission settings, as it is these gametocytes that prevent complete elimination of the disease.

Artemisinin combination therapy (ACT) combined with primaquine is the only transmission blocking therapy currently available for uncomplicated malaria by *P. falciparum* (WHO Guidelines for Malaria 2023). However, increased resistance to artemisinin in geographical regions like Southeast Asia highlights the need for therapeutic approaches to prevent persistence of parasites in the bloodstream conferring transmission (Stokes et al. 2021). The low efficacy of the vaccine (RTS,S/AS01) for children, recently recommended by the World Health Organization (WHO), also indicates the need for ideal transmission blocking vaccine (TBV) for long-term reduction in transmission offering immunity against epidemics if the disease were to resurface.

Gametocytes exhibit unique expression patterns and are highly conserved, with minimal genetic variations as compared to asexual stages. Additionally, gametocyte-specific proteins induce the production of antibodies in naturally infected individuals, leading to significant reduction in transmission. These inherent characteristics of gametocytes position them as promising targets for the development of effective TBVs (Dantzler et al. 2019). Developing effective interventions against the transmission of *P. falciparum* requires a comprehensive understanding of the molecular mechanism and pathology of the parasite’s sexual stages, as well as exploring the naturally acquired human immune response directed against these stages to guide the formulation of a robust TBV (Jong et al. 2020; Chandley et al. 2023). Since, only a small number of sexual stage vaccines (P25, P28, P48/45, P230, P47) have been tested in preclinical investigations, novel strategies to enhance TBV research and development, particularly novel candidate discovery, are required. The abundance of transcriptomic data available from various studies on *P. falciparum* can provide critical inputs allowing for the identification of potential biomarkers for transmission and markers specific to transmission stage and conditions, enabling the development of interventions targeting the genes.

The purpose of this review is to comprehensively examine transcriptomic datasets related to *P. falciparum* sexual stages and identify the genes specific to gametocyte transmission from humans to mosquitoes, which is necessary to complete the parasite’s life cycle. The article aims to demonstrate how publicly available large volumes of transcriptomic datasets can be utilized to investigate unexplored genes and key genes involved in transmission. Additionally, the study identified the expression levels of potential genes from the publicly available RNA-Seq datasets and reviewed their functions associated with transmission, based on the current knowledge in the field.

## Exploring the genes regulating *P. falciparum* transmission

**Fig. 1:**
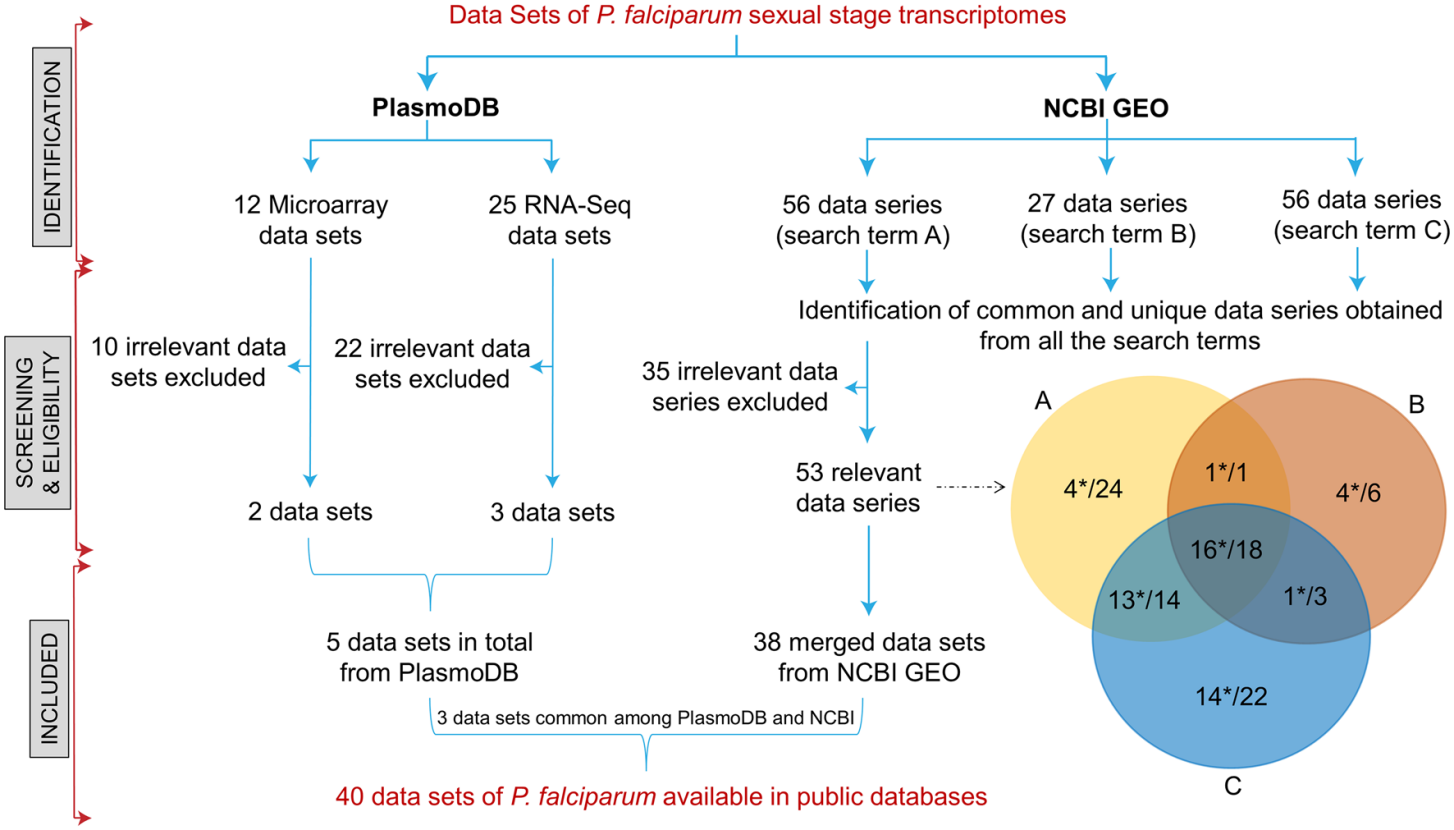
Workflow depicting the process of identifying transcriptomic datasets from publicly available databases. The search terms used in NCBI-GEO to identify datasets: A: ‘*P. falciparum* AND transmission’, B: ‘*P. falciparum* AND sexual stages’, C: ‘*P. falciparum* AND gametocytes’ (datasets relevant are denoted by * in the Venn diagram)

Transcriptomic datasets related to *P. falciparum* sexual stage development and transmission were retrieved from the publicly available PlasmoDB and NCBI-GEO databases. The search terms ‘*P. falciparum* AND transmission’, ‘*P. falciparum* AND sexual stages’, and ‘*P. falciparum* AND gametocytes’ were used to search and identify the potential RNA-Seq datasets. The flowchart (Fig. 1) clearly and visually depicts the process of identifying datasets. A total of 40 RNA-Seq datasets were identified and grouped together based on their similar topics of focus—(1) epigenetics and chromatin (*n* = 10); (2) transcriptional regulators (*n* = 7); (3) early gametocyte stage (*n* = 6); (4) mid and late gametocyte stage (*n* = 11); (5) effects of inhibitors on gametocytes (*n* = 6). Variations in sample type, study design and platform technique were observed among the identified datasets. Most of the datasets were generated using in vitro* P. falciparum* infected red blood cells (iRBCs), with a total of 35 datasets with three exceptions where two datasets used whole blood sample and one dataset used mosquito midgut sample (Supplementary Table 1).

**Fig. 2:**
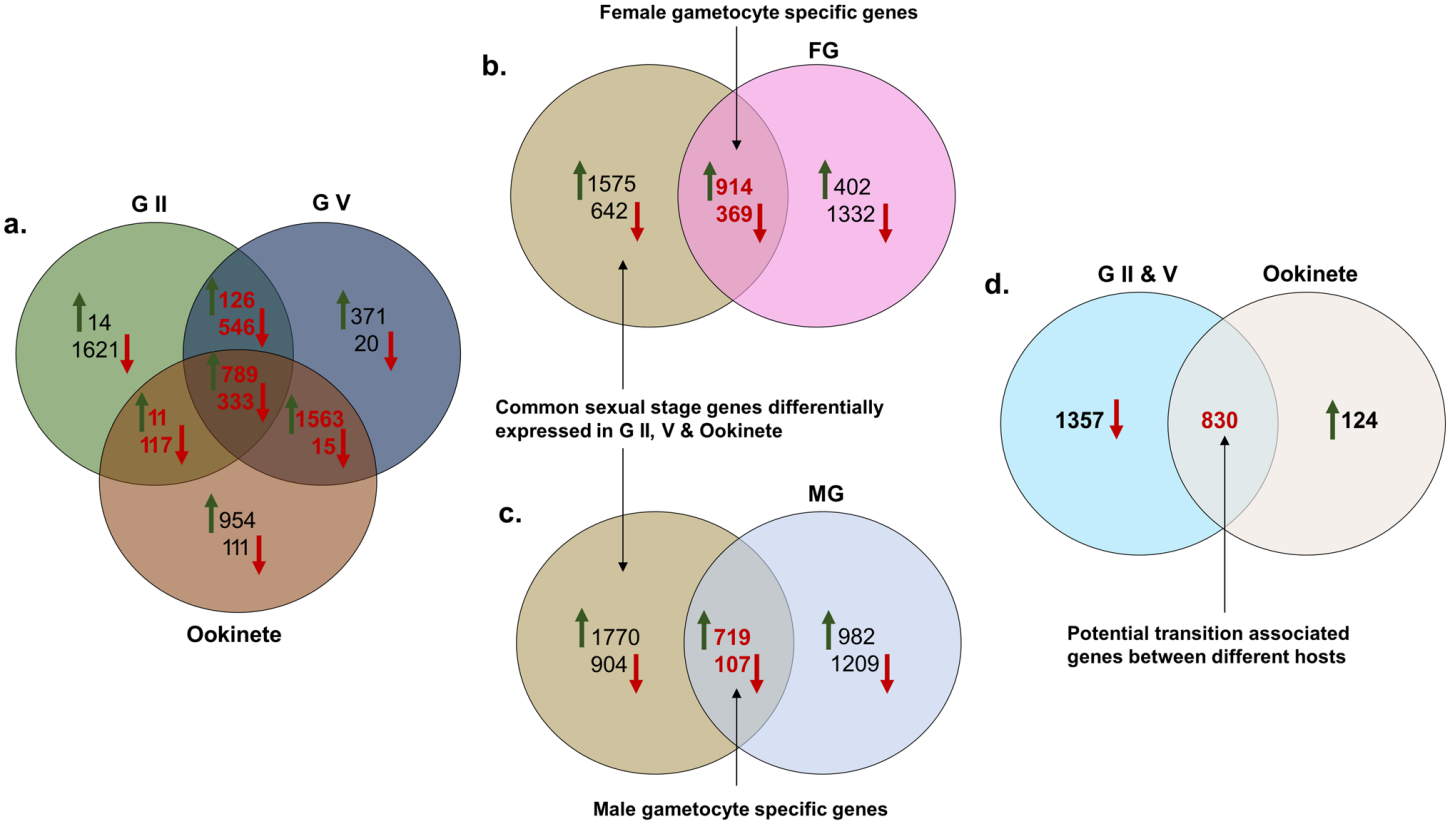
Venn diagrams depicting: common and unique genes expressed among gametocyte stage II, V, and ookinete (**a**); sexual stage specific and female gametocyte differentially expressed genes identifying common genes expressed throughout female gametocyte development (**b**); sexual stage specific and male gametocyte differentially expressed genes identifying common genes expressed throughout male gametocyte development (**c**); downregulated genes in gametocyte stage II and V occurring in humans with upregulated genes in ookinete occurring in mosquito vector identifying potential transition associated genes during transmission (**d**). *G II* gametocyte stage II, *G V* gametocyte stage V, *FG* female gametocyte, *MG* male gametocyte

In this study, two RNA-Seq datasets available in PlasmoDB, referred to as SRP009370 (Lasonder et al. 2016) and GSE75795 (López-Barragán et al. 2011), were chosen to examine the gene expression levels across sexual stages of *P. falciparum*. The SRP009370 dataset provided information on gene expression levels in both asexual and sexual stages, while the GSE75795 dataset provided information on gene expression levels in male and female gametocytes. Differential expression analysis (DEA) was performed on both datasets (Supplementary File 1, Supplementary Table 2) and the results were used to generate Venn diagrams (Fig. 2a–d) which provided information on the number of genes that might be involved in transmission. Through DEA, a total of 3500 (2489 upregulated and 1011 downregulated) common genes across gametocyte stage II, V, and ookinete stages were identified. 1283 (914 upregulated and 369 downregulated) genes were found to be common to female gametocytes, while 826 (719 upregulated and 107 downregulated) genes were found to be common to male gametocytes. Out of the comprehensive set of common genes, only a few genes have established functions in transmission, whereas remaining genes require further exploration to determine their roles. Additionally, we identified 830 genes that exhibit differential expression during the transition of the parasite stage from the human host to mosquito host. This differential regulation suggests their involvement in vital processes crucial for the parasite’s adaptation and survival in diverse hosts, making them potential TBV candidates. Further exploration of these genes holds promise for gaining valuable insights into the parasite’s lifecycle and its intricate interactions with both human and mosquito hosts.

## Exploring the potential of genes with known functions as TBV candidates

**Fig. 3:**
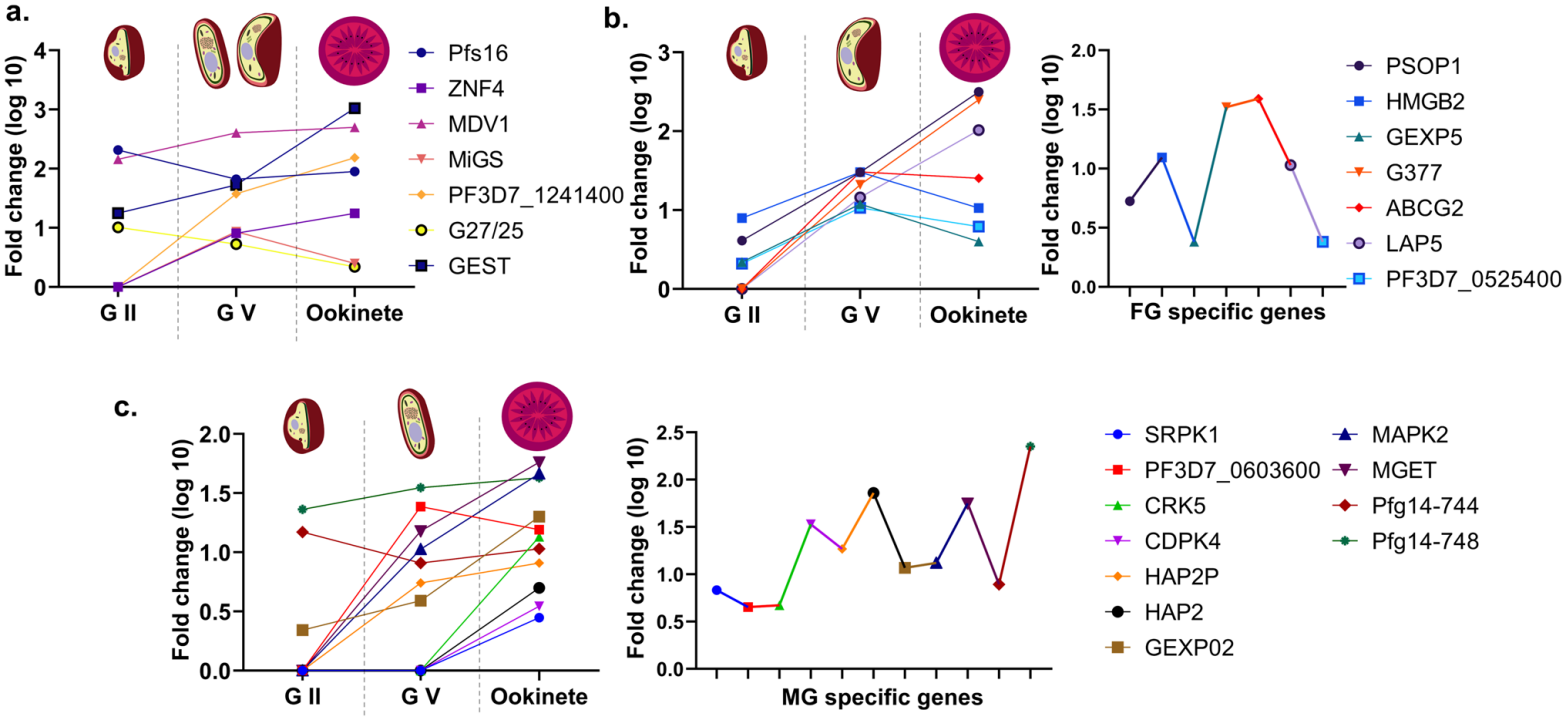
Line plots depicting expression levels of *P. falciparum* gametocyte genes with known role in transmission obtained from published literature (cited in the review). The expression levels of genes expressed in both the sex gametocytes in gametocyte stage II, V, and ookinete (**a**); the expression levels of female and male specific gametocyte genes in gametocyte stage II, V, and ookinete, respectively (**b**, **c**). Additionally, the relative expression levels are presented to highlight the genes that are expressed at higher or lower levels (**b**, **c**). The expression level values were obtained from the RNA-Seq datasets SRP009370 and GSE75795. *G II* gametocyte stage II, *G V* gametocyte stage V, *FG* female gametocyte, *MG* male gametocyte

The *P. falciparum* reference genome (GCA_900632045.1) is 2.33 Mb in size with GC content of 19.33%, and the genome consists of 5383 protein coding genes. The genes *Pfs25*, *Pfs28*, *Pfs48/45*, *Pfs230*, and *Pfs47* are being investigated as potential targets for TBVs and only a phase I/II trial is currently ongoing (Chaturvedi et al. 2016). In addition, other gametocyte genes implicated in transmission through genetic evidence may also provide candidates for such vaccines (Table 1). To better understand the role of these genes in transmission, their expression levels in various sexual stages were analysed using RNA-Seq datasets SRP009370 and GSE75795 (Supplementary Table 3, Fig. 3a–c).

**Table 1:** List of potential transmission blocking vaccine candidate genes and their functions

Gene ID	Gene symbol	Function	References
PF3D7_0302100	*SRPK1*	Involved in pre-mRNA splicing and male gamete fertility	Kumar et al. (2022a)
PF3D7_0406200	*Pfs16*	Initiates gametocytogenesis and maintains parasitophorous vacuolar membrane integrity	Yahiya et al. (2023)
PF3D7_0525400	N/A	Involved in maintaining stress granule function and formation of ookinete	Bennink et al. (2018)
PF3D7_0603600	N/A	Involved in exflagella formation in male gametocytes	Kumar et al. (2022d)
PF3D7_0615500	*CRK5*	Regulates DNA replication and exflagella formation in male gametocytes	Kumar et al. (2022b)
PF3D7_0717500	*CDPK4*	Essential for the development of flagellated microgametes and their separation from activated microgametes	Kumar et al. (2021)
PF3D7_0721700	*PSOP1*	Facilitates gamete emergence	Ishino et al. (2020)
PF3D7_0816300	*HAP2P*	Facilitates gamete fusion	Kumar et al. (2022c)
PF3D7_0817900	*HMGB2*	Facilitates sexual phase development and regulates sporozoite production	Kumar and Kappe (2022)
PF3D7_0936600	*GEXP5*	Involved in early gametocyte development	Barry et al. (2021)
PF3D7_1014200	*HAP2*	Involved in male gamete fertility and fusion	Angrisano et al. (2017)
PF3D7_1102500	*GEXP02*	Facilitates host cell remodelling during gametocyte development	Warncke et al. (2020)
PF3D7_1113900	*MAPK2*	Plays a role in male gametogenesis	Hitz et al. (2020)
PF3D7_1134600	*ZNF4*	Involved in exflagella formation in male gametocytes	Hanhsen et al. (2022)
PF3D7_1216500	*MDV1*	Involved in exflagella formation in male gametocytes	Tripathi et al. (2021)
PF3D7_1234400	*MiGS*	Involved in gamete fusion	Grasso et al. (2022)
PF3D7_1241400	N/A	Associated with female gamete formation	Kumar et al. (2022e)
PF3D7_1250100	*G377*	Important for formation of female gametocyte osmiophillic bodies	Ishino et al. (2020)
PF3D7_1302100	*G27/25*	Involved in early gametocyte development	Tibúrcio et al. (2015)
PF3D7_1426500	*ABCG2*	Essential for female gametocyte and zygote formation	Tran et al. (2014) and Kenthirapalan et al. (2020)
PF3D7_1449000	*GEST*	Facilitates gamete emergence	Ishino et al. (2020)
PF3D7_1451600	*LAP5*	Essential for ookinete formation	Tremp et al. (2017) and Obaldía et al. (2022)
PF3D7_1469900	*MGET*	Involved in male gametocyte formation	Stone et al. (2017) and Bradley et al. (2018)
PF3D7_1477300	*Pfg14-744*	Involved in early gametocyte development	Eksi et al. (2005)
PF3D7_1477700	*Pfg14-748*	Involved in early gametocyte development	Eksi et al. (2005)

N/A: Not Available

## Gametocyte gene involved in maintaining integrity and stability of PVM

*Plasmodium* utilizes an invagination mechanism to enter the host RBCs, forming a parasitophorous vacuolar membrane (PVM) surrounding the parasite and undergo modification facilitated by its secretory organelles. The PVM acts as a barrier that separates the parasite from the host cell’s lysosomal machinery, preventing parasite’s destruction (Goldberg and Zimmerberg 2020). The expansion of the PVM is a critical process for the growth and maturation of *P. falciparum* within the RBCs, which lack internal organelles and are no longer capable of active membrane synthesis. *Pfs16*, encoding a member of the early transcribed membrane proteins (ETRAMPs) family, is an early marker for sexual commitment and believed to play a key role in maintaining PVM integrity and stability. Studies have demonstrated that *Pfs16* has a dual role and even its truncated versions can initiate gametocytogenesis. Pfs16 is comprised of two distinct domains: an N-terminal domain responsible for secretion to the parasitophorous vacuole (PV), and a C-terminal domain that facilitates association with the PVM. The PVM domain consists of a transmembrane domain and a 31-amino acid long C-terminal tail, in which last 11 amino acids playing a critical role in enhancing Pfs16 retention on the membrane. In addition, the region between the N-terminal and transmembrane domains is essential for capping (Eksi and Williamson 2011). In a recent study, the use of an N-[(4-hydroxychroman-4-yl) methyl]-sulphonamide (N-4HCS) compound targeting Pfs16 has demonstrated its critical role in the earliest stages of micro gametogenesis, where the PVM remains associated with the parasite (Yahiya et al. 2023). This finding was further validated by observing an increase in its expression fold change value during the ookinete stage (Fig. 3a).

## Genes involved in shaping, remodelling, and regulating behaviour of gametocytes

Two decades ago, researchers found genes specific to the *P. falciparum* sub-telomeric superfamily expressed in parasites during sexual development, evidenced by co-expression of the well-known early gametocyte stage proteins Pfs16 and Pfg27. Despite this discovery, the specific role of these sub-telomeric superfamily genes in transmission control have not been studied since. The gene family includes two members, *Pfg14-748* and *Pfg14-744*, with distinct expression patterns associated with *Pf*AP2-G binding. *Pfg14-748* begins to express in the final stages of schizont just before the release of merozoites committed for gametocytogenesis. On the other hand, *Pfg14-744* starts to express once the parasite invades and the newly formed ring begins the process of sexual differentiation. Both the proteins are expressed in gametocyte committed ring stage parasites and are localized in the RBCs and the PV, respectively, and play a role in creating a specific environment for gametocyte development, giving it a unique shape and behaviour compared to other iRBCs (Eksi et al. 2005). In contrast to previous reports, analysis of RNA-Seq data has revealed persistent expression of these genes not only in early gametocytes, but throughout the sexual stages, with particularly elevated expression in male gametocytes (Fig. 3c).

Gametocyte exported proteins (*GEXPs*) are members of the *Plasmodium* helical interspersed sub-telomeric (PHIST) protein family, which are expressed in both asexual and sexual stages of the parasite. However, certain GEXPs are exclusively present in gametocytes, *GEXP5*, an AP2-G-independent gametocyte specific gene is overexpressed in the cytoplasm of ring stage parasites committed to gametocytogenesis. GEXP5 is absent in asexual ring stages, making it a potential marker for early-stage of gametocyte development (Barry et al. 2021). A study has shown that *Pf*GEXP5 expression occurs prior to the well-known early gametocyte development protein Pfg27 (Tibúrcio et al. 2015) and is sustained throughout the sexual stages, particularly overexpressed in female gametocytes (Fig. 3b). Inhibition of PVM prevents GEXP processing and export, which plays a role in preparing the GEXPs for export in gametocytes, ultimately obstructing gametocyte differentiation stages II-V (Jennison et al. 2019). Although GEXP5 is currently only utilized as a marker for detecting circulating gametocyte-ring stages and quantifying sexual conversion rates, its exact function in female gametocyte development and transmission remains to be further explored.

GEXP02, another PHIST protein is highly expressed in male gametocytes, responding at the earliest to *PfAP2-G* activation. It is localised in gametocyte-infected erythrocyte (GIE) membrane and is involved in remodelling of host cells during gametocytogenesis, making it an important candidate for host–parasite protein interaction (Warncke et al. 2020). Despite having higher expression levels than *PfAP2-G*, it is yet to be established if GEXP02 can be used as a marker for epidemiological investigations (Portugaliza et al. 2019). Previous studies have suggested that *GEXP02* is not necessary for the development of gametocytes, transmission to mosquitoes, production of sporozoites, or hepatocyte infection. However, its high expression in the ookinete stage (Fig. 3c) indicates that it may play a critical role in sexual stages in mosquitoes, which requires further investigations.

## Gametocyte genes associated with adaptation

Living organisms possess proteins that aid in their adaptation to changes in their environment, such as light, water, temperature, and others. The acquisition of *P. falciparum* gametocytes by the mosquito vector leads to significant alterations in the environment, such as a decrease in temperature, an increase in pH, and the presence of mosquito midgut factors, e.g., xanthurenic acid (Dash et al. 2022), necessitating the synthesis of adaptation proteins. These proteins are produced within the stress granules (SGs) present in the female gametocytes and play a critical role in ensuring the survival and transmission of the parasite. Despite the importance of SGs in the transmission of the parasite, the molecular components and regulatory mechanisms involved in their function are not fully understood. A recent discovery of the PF3D7_0525400 (7-helix-1) transcript, a homolog of stress response regulator in humans *LanC-like 2*, encoding a protein crucial for mosquito infectiousness, has provided new insights. PF3D7_0525400 was found to interact with translational repressors to maintain proper SG function and associates with mRNA that codes for female-specific antigen Pfs25, thereby impacting its synthesis and ookinete formation. Additionally, researchers also revealed that the loss of *PF3D7_0525400* results in a transcriptional deregulation of proteins required for translational control and down-regulation of non-coding RNAs required for formation and operation of ribosomes (Bennink et al. 2018).

Another such protein is the ABCG2, ATP-binding cassette (ABC) transporter, which is present in all the *Plasmodium* species and performs different functions depending on the host environment. This protein is a neutral lipid transporter exclusively expressed in female gametocytes and is important for membrane biogenesis and hemozoin formation in parasite’s digestive vacuole. It is also involved in the formation of zygote from gametes in the female mosquitoes, highlighting its significance in transmitting the parasite to the mosquito vector. The *ABCG2* was knocked-out in a study, leading to increased gametocyte commitment and different protein localization in different *Plasmodium* species. The replaced gene from the alternate species did not restore normal gametocyte numbers, suggesting that similar proteins can have different functions and targeting these could aid in developing better vaccines (Tran et al. 2014; Kenthirapalan et al. 2020). RNA-Seq data analysis shows that *PF3D7_0525400* and *ABCG2* are expressed throughout sexual stages, indicating their crucial role in the modulation of environmental changes experienced by gametocytes during their journey from the bone marrow to their eventual arrival in the blood circulation and further transit to the mosquito midgut for the continuation of the sexual stage process (Fig. 3b).

## Genes associated with gametocyte activation

After the uptake of gametocytes by the mosquito during a blood meal, osmiophilic bodies (OBs), which are specialised secretory vesicles found solely in gametocytes help the gametocyte to escape from the iRBCs. OBs are dispersed in the gametocyte’s cytoplasm, which then move towards the inner membrane complex, and merge followed by exocytosis of the fused female OBs leading to breakdown of PVM and erythrocyte membrane allowing gametocyte egress. The gamete egress and sporozoite traversal (*GEST*), *Plasmodium* secreted ookinete protein (*PSOP1*), male development gene 1 (*MDV1*), and *G377* encodes protein localised to OBs and are released during gamete emergence. *PfG377* is overexpressed in female gametocyte OBs (Fig. 3b) crucial for its formation, but not for parasite egress. It is highly variable among parasite population, which allows many parasite lines to unite and persist at the same time, resulting in transmission dynamics (Ishino et al. 2020). A recent study aimed to determine the essentiality of *Pf*MDV1 for successful transmission to mosquitoes reported that *Pf*MDV1 exhibits low antigenic variation and are expressed in the inner membrane pellicle complex from stage III-V throughout gametocytogenesis. MDV1 play a critical role in female gametocyte activation and accumulates in a few foci in the activated male gametocyte which are maintained until exflagellation. Additionally, *Pf*MDV1 generates naturally occurring antibodies in the adult population of Ghana, hinting that targeting these antigens could result in long-lasting immune responses (Tripathi et al. 2021). Recently, researchers identified a new protein called microgamete surface protein (MiGS) which is expressed in both the sex gametocytes of *P. falciparum* but is upregulated specifically in male gametocytes. MiGS showed a punctate signal in both sexes, but in activated gametocytes, it moved to the cell periphery. This indicates that male gametocytes of *P. falciparum* possess secretory organelles that respond to signals associated with the process of gametogenesis (Grasso et al. 2022). In *P. berghei* and *P. yoelii*, MiGS was found only in male gametocytes and was shown to interact with MDV1. The presence of *Py*MiGS on the surface of the microgamete implies that it could have a function in fertility such as recognition, attachment, or fusing with viable female gametes (Tachibana et al. 2018). Hence, these functions still need to be explored in *P. falciparum*.

## Male gamete fertility associated genes

Kinases, such as *Pf*MAP-1, *Pf*MAP-2, *Pf*SRPK1, *Pf*CDPK4, and *Pf*CRK5 are critical regulators of signalling pathway and play a vital role in different stages of the malaria parasite’s life cycle. *Pf*MAP-1 and *Pf*MAP-2 are the only two homologues of eukaryotic mitogen-activated protein kinases (MAPKs) are found in *Plasmodium*. They play a crucial role in cellular signaling, cell growth and differentiation during gametogenesis and have unique features. *Pf*MAP-2 is particularly essential for exflagellation, genome condensation and axoneme beating. Although microarray experiments did not identify any transcriptional changes linked to *PfMAPK2*'s  function, suggesting its role in male gametogenesis associated with phosphorylation-mediated alterations in target substrate activity rather than regulation of gene expression (Hitz et al. 2020). The crucial kinase, *Pf*SRPK1, responsible for pre-mRNA splicing and male gamete fertility is present in both the nuclear compartments and cytoplasm of *Plasmodium*, but its function is particularly noteworthy in the cytoplasm (Kumar et al. 2022a). Investigating the role of *SPRK* in chromatin states in other organisms (Gou et al. 2020) could offer valuable insights into its similar function in *P. falciparum*. Calcium-dependent protein kinases 4 (CDPK4), another important kinase in male gamete fertility, is essential for the development of flagellated microgametes that are capable of separating and exiting from activated microgametes. This kinase is localized in the membrane with cytoplasmic staining and expressed in both the asexual and sexual stages of *P. falciparum* (Kumar et al. 2021). Additionally, *Pf*CRK5 localized in peri-nuclear region, cytoplasm, and membrane of male gametocytes is necessary for the regulation of DNA replication and formation of exflagella during male gametogenesis (Kumar et al. 2022b).

The class II viral fusion proteins belonging to HAP2/GCS1 family members are critical for male gamete fertility. *P. falciparum* has two HAP2/GCS1 family members that mediate cell membrane fusion independently, making them authentic cell/gamete fusogens. When activated, a hydrophobic region (cd-loop) located in the HAP2 proteins displays itself on the plasma membrane of the male gamete and enters the plasma membrane of the female gamete while creating trimers, surmounting the energetic obstacles that hinder membrane fusion. The expression of *PfHAP2* occurs during the development of male gametocytes and in male microgametes, whereas the expression of *PfHAP2P* takes place in the late-stage V gametocytes and microgametes (Fig. 3c). The male gamete uses these fusogens solely to facilitate the fusion process with the female gamete. It is not evident whether they function in a unidirectional or bidirectional manner and whether they serve individual and distinct functions or work together in a complex. The mechanisms of the signalling events that trigger the precise surface positioning and activation of *PfHAP2* and *PfHAP2P* at the appropriate time are currently unknown and require further investigation (Angrisano et al. 2017; Kumar et al. 2022c). Also, a study has demonstrated the efficacy of a self-assembling protein nanoparticles (SAPN) vaccine based on *Pf*GCS1 antigenic determinants and universal T-helper epitopes in enhancing immunogenicity, through in silico and in vitro methods (Zahedi et al. 2022).

Chromatin remodelling is necessary to regulate the expression of specific genes during *Plasmodium* gametocytogenesis and gametogenesis. While the ApiAP2 family of transcription factors regulate gametocytogenesis, the remodelling complexes involved in gametogenesis are poorly characterised in *P. falciparum*. Recently, PF3D7_0603600, the AT-rich interaction domain (ARID), a DNA-binding domain involved in eukaryotic transcriptional processes was identified and has been validated to play a role in male gametogenesis in *P. falciparum*. PF3D7_0603600 colocalizes with H3K9Ac in the nucleus, suggesting its involvement in regulating gene expression during the sexual stage process in the mosquito. Although *PF3D7_0603600* is expressed in both asexual and sexual stages, knockout studies showed it is not essential for asexual parasite survival or gametocyte development. However, it is critical for flagellated microgamete formation after gametocytes are taken up by the vector (Kumar et al. 2022d). Similarly, ZNF4, a member of CCCH zinc finger proteins expressed in both the sex gametocyte’s cytoplasm is essential for only male gamete fertility specifically playing crucial role in male gametocyte exflagellation (Hanhsen et al. 2022). From RNA-Seq data analysis, male gametocyte-enriched transcript (*Pf*MGET) showed a high level of expression in mature gametocyte stage, particularly in male gametocytes (Fig. 3c). It has been used as a marker that is specific to assess the abundance of circulating male gametocytes in infected individuals (Stone et al. 2017; Bradley et al. 2018). Despite its conspicuous expression pattern, the functional relevance of *PfMGET* in transmission has not been investigated yet.

## Female gamete fertility associated genes

The *Plasmodium* genome encodes numerous RNA binding proteins, but their roles in gamete fertility were unknown until recently. PF3D7_1241400, Macrogamete Contributed Factor Essential for Transmission (MaCFET), a putative RNA binding protein is essential for transmission and expressed at the highest levels in gametocytes and localizes to the cytoplasm. It is expressed in both the sex gametocytes but specifically required for regulating female gamete function, although the reason for this sex-specificity is still unknown (Kumar et al. 2022e). Another gene encoding for the high-mobility group box (*HMGB*), previously known to induce pro-inflammatory cytokine in humans (Kumar et al. 2008), is expressed in both asexual and sexual stages of the parasites in human host, does not play a necessary role for their development. However, knockout studies have shown that it does have a modest impact on the sexual phase development of the parasite by reducing the number of sporozoites produced in the mosquito. While the HMGB may not be essential for the parasite to complete its sexual cycle, it may still be critical for its transmission to a new host. While HMGB2 is involved in male fertility and spermatogenesis in turtles, it is not essential for *Plasmodium* male gamete development (Kumar and Kappe 2022). Analysis of RNA-Seq data (Fig. 3b) suggests that HMGB2 may play a significant role in female gamete fertility, which requires further investigation.

## Ookinete development associated genes

LCCL proteins are a type of modular proteins that are unique to apicomplexan parasites. *Plasmodium* has six members of this protein family called LCCL lectin adhesive-like proteins (LAP) 1–6 in *P. berghei*. Recent studies have shown that LAPs are crucial for the formation of crystalloids, which are transient subcellular organelles present only in ookinetes and young oocysts and are associated with malaria transmission. LAP5, a member of the LAP protein complex, has been extensively studied in *P. berghei* and has been identified as a suitable marker for determining the human transmission reservoir of *P. vivax* using molecular assays. *PfFNPA*, an ortholog of *LAP5* present in *P. falciparum*, is expressed in mature female gametocytes as well as later stages of gametogenesis. Understanding the molecular interactions of FNPA is crucial, as interfering with its interactions could potentially lead to the blockade of malaria transmission (Tremp et al. 2017; Obaldía et al. 2022).

## Limitations and knowledge gaps

The scope of this review was restricted to transcriptomic datasets exclusively related to *P. falciparum*, as most of the data available on gametocyte biology studies predominantly focused on this species. Consequently, the potential candidates identified in *P. falciparum* could be further evaluated by comparing them with orthologs of *P. vivax* and other human-infecting *Plasmodium* species like *P. malariae*, *P. ovale*, or *P. knowlesi*. This comparative approach could potentially reveal common candidates associated with human-to-mosquito transmission, serving as potential universal targets for TBVs across all five human-infecting *Plasmodium* species. However, the analysis was based on a limited number of RNA-Seq datasets, and to enhance the robustness of the conclusions, it would be beneficial to perform multiple dataset analysis. Integrating proteomic approaches could offer a more comprehensive understanding of the candidate genes, providing valuable insights into translation efficiency and identifying potentially translationally repressed transcripts, thus further narrowing down the candidate list.

Most of the studies on *Plasmodium* gametocytogenesis utilize the data derived from in vitro cultured gametocytes rather than expression data derived directly from human-infected samples. Thus, there is a knowledge gap in assessing the expression of important TBV candidates in natural infections, as it remains unclear whether the expression of TBV candidate genes varies under the following situations: (1) different malaria-endemic regions with varying transmission settings, such as seasonal malaria or high/low transmission intensities; (2) different ethnic populations, age groups, immunity levels, and disease severities; (3) imported malaria cases involving asymptomatic travellers in non-endemic settings, among others. Addressing these limitations and knowledge gaps through studies involving natural infections and diverse population contexts would significantly contribute to a more comprehensive understanding of transmission biology and aid in the development of effective TBVs.

## Conclusion

The high throughput RNA-Seq approach is widely used to profile the complete set of transcripts in a cell. Further, advancements in information technologies provided an opportunity to identify the potential genes responsible for various biological activities and host–pathogen interactions. Hence, the study demonstrates the appropriate usage of publicly available transcriptomic datasets, internet-oriented data analytics, and thorough literature to explore the key genes playing crucial role in malaria parasite transmission from humans to mosquitoes. In conclusion, our review highlights the importance of utilizing publicly available transcriptomic datasets to better understand the biology of *P. falciparum* gametocytes. By analysing relevant datasets, we demonstrated how this approach can be effective in identifying genes associated with sexual stage development, which could be explored for their roles in transmission and targeted for TBVs. Additionally, we reviewed the functions of known gametocyte genes, identifying their expression patterns throughout sexual stages to improve our understanding of gametocyte biology. The comprehensive review on transcriptomic data and literature may reveal new opportunities for developing innovative strategies to control malaria transmission.

### Supplementary Information

Below is the link to the electronic supplementary material.

Supplementary Table1 (XLSX 24 KB)

Supplementary Table2 (XLSX 1482 KB)

Supplementary Table3 (XLSX 16 KB)

Supplementary File 1 (DOCX 22 KB)

## Data Availability

All data generated during this study are included in this article and its supplementary information files.
