# Supplementary material for: Transcriptomic approaches for identifying potential transmission blocking vaccine candidates in Plasmodium falciparum: a review of current knowledge and future directions
Source: 3 Biotech. 2023 Sep 12;13(10):344. doi: 10.1007/s13205-023-03752-3 (PMC10497465; doi:10.1007/s13205-023-03752-3)
Supplement: Supplementary file 4 — Supplementary File 1 (DOCX 22 KB) [file 13205_2023_3752_MOESM4_ESM.docx]

**3 Biotech**

**Transcriptomic approaches for identifying potential transmission blocking vaccine candidates in *Plasmodium falciparum*: A review of current knowledge and future directions**

Gutthedhar Varijakshi^1^, Mallya Divya^1^, Akshay Pramod Ware^2^, Bobby Paul^2^, and Abdul Vahab Saadi^1,*^

^1^Department of Biotechnology, Manipal School of Life Sciences, Manipal Academy of Higher Education, Manipal 576104, Karnataka, India

^2^Department of Bioinformatics, Manipal School of Life Sciences, Manipal Academy of Higher Education, Manipal 576104, Karnataka, India

*Corresponding author: [saadi.a@manipal.edu](mailto:saadi.a@manipal.edu)

**Supplementary File 1** Methodology for performing DEA on RNA-Seq datasets using Venn diagrams**.**

1. We selected SRP009370 and GSE75795 datasets from PlasmoDB for our analysis. SRP009370 comprised of gene expression data for four asexual stages (sporozoite, ring, early and late trophozoites) and sexual stages (gametocyte stage II, V, and ookinete) of *P. falciparum*. And GSE75795 comprised of gene expression data for male and female gametocytes of *P. falciparum*.
2. Using SRP009370 dataset, we performed DEA to identify genes that exhibit differential expression between all four asexual stages combined with individual sexual stages. The analysis results are summarized in Table 1 below. The corresponding supplementary information (Tables 2a-c) represents these results.

**Table 1 Summary of DEA for SRP009370 dataset for all asexual stages with each sexual stage**

| **Method** | | **Results (No. of genes)** | | | **Supplementary reference** |
| --- | --- | --- | --- | --- | --- |
| **Reference sample** | **Comparison sample** | **Upregulated** | **Downregulated** | **Total** |  |
| Asexual stages | Gametocyte stage II | 940 | 2617 | 3557 | 2a |
| Asexual stages | Gametocyte stage V | 2849 | 914 | 3763 | 2b |
| Asexual stages | Ookinete | 3317 | 576 | 3893 | 2c |

1. The total differentially expressed genes obtained from step 2 for each sexual stage were subjected to Venn analysis. This analysis aimed to identify the unique and common genes among the three sexual stages, which are depicted in the Venn diagram (Fig. 2a) of the main manuscript, supplementary information (Tables 2d-2k) and summarized in Table 2 below.

**Table 2 Summary of Venn analysis to identify common and unique genes**

| **Parasite stage** | **Results (No. of genes)** | | | **Supplementary reference** |
| --- | --- | --- | --- | --- |
|  | **Upregulated** | **Downregulated** | **Total** |  |
| **Common differentially expressed genes** | | | | |
| Gametocyte stage II & V | 126 | 546 | 672 | 2d |
| Gametocyte stage II & ookinete | 11 | 117 | 128 | 2e |
| Gametocyte stage V & ookinete | 1563 | 15 | 1578 | 2f |
| Gametocyte stage II, V & ookinete | 789 | 333 | 1122 | 2g |
| **Total** | 2489 | 1011 | 3500 | 2h |
| **Unique differentially expressed genes** | | | | |
| Gametocyte stage II | 14 | 1621 | 1635 | 2i |
| Gametocyte stage V | 371 | 20 | 391 | 2j |
| Ookinete | 954 | 111 | 1065 | 2k |
| **Total** | 1339 | 1752 | 3091 |  |

1. Using GSE75795 dataset, we performed DEA to identify genes that exhibit differential expression in female gametocyte when compared with male gametocyte (Upregulated: 1316, Downregulated: 1701, Total: 3017) and male gametocyte when compared with female gametocyte (Upregulated: 1701, Downregulated: 1316, Total: 3017). The corresponding supplementary information (Table 2l) represents these results.
2. Venn analysis was conducted between the common differentially expressed genes (3500) obtained from step 3 and differentially expressed genes of male and female gametocytes (3017) obtained from step 4. This analysis aimed to identify genes that are unique to each gender and expressed throughout the gametocyte development. The results are depicted in Venn diagrams (Fig. 2b, c) in the main manuscript, while Table 3 provides a summary of the findings. For a comprehensive understanding of the identified genes, the exhaustive gene list is presented in the supplementary information (Tables 2m, n).

**Table 3 Summary of Venn analysis to identify sex-specific differentially expressed genes**

| **Gametocyte stage** | **Results (No. of genes)** | | | **Supplementary reference** |
| --- | --- | --- | --- | --- |
|  | **Upregulated** | **Downregulated** | **Total** |  |
| **Common differentially expressed genes** | | | | |
| Female gametocyte | 914 | 369 | 1283 | 2m |
| Male gametocyte | 719 | 107 | 826 | 2n |
| **Unique differentially expressed genes** | | | | |
| Female gametocyte | 402 | 1332 | 1734 |  |
| Male gametocyte | 982 | 1209 | 2191 |  |

1. To identify potential transition associated genes during transmission of *P. falciparum* gametocytes, we conducted a Venn analysis between downregulated genes in sexual stages occurring in humans (gametocyte stage II: 1621, gametocyte stage V: 20, gametocyte stage II & V: 546) and the upregulated genes in sexual stages occurring in mosquito vector (ookinete: 954) obtained from step 3. The results of this analysis are visualized in the Venn diagram (Fig. 2d) included in the main manuscript. For a comprehensive list of the identified genes, refer to the supplementary information (Table 2o).
